# Comparison of CYP2C9 activity between Ethiopian and non-Ethiopian Jews: an interethnic study of (*S*)-warfarin pharmacokinetic and pharmacodynamic

**DOI:** 10.3389/fphar.2026.1836874

**Published:** 2026-06-23

**Authors:** Maor Wanounou, Zahi Abu Ghosh, Chanan Shaul, Waseem Mujahed, Shoshana Alamia, Simcha Blotnick, Meir Bialer, Yoseph Caraco

**Affiliations:** 1 Clinical Pharmacology Unit, Division of Medicine, Hadassah Medical Center, Faculty of Medicine, Hebrew University of Jerusalem, Jerusalem, Israel; 2 Institute of Drug Research, School of Pharmacy, Faculty of Medicine, Hebrew University of Jerusalem, Jerusalem, Israel; 3 Heart Institute, Hadassah Medical Center, Faculty of Medicine, Hebrew University of Jerusalem, Jerusalem, Israel; 4 Department of Otolaryngology and Head and Neck Surgery, Shaare-Zedek Medical Center, Faculty of Medicine, Hebrew University of Jerusalem, Jerusalem, Israel; 5 Department of Emergency Medicine, Hadassah Medical Center, Faculty of Medicine, Hebrew University of Jerusalem, Jerusalem, Israel

**Keywords:** (S)-warfarin, CYP2C9 activity, interethnic variability, pharmacodynamics, pharmacokinetics

## Abstract

**Introduction:**

CYP2C9 is characterized by marked interindividual variability. A significant fraction of this variability is attributed to ethnic specific genetic polymorphisms. We have recently shown that the activity of CYP2C9 as measured by phenytoin metabolic ratio is decreased by 30% among Ethiopians as compared to non-Ethiopians Jews carriers of CYP2C9*1/*1 genotype. The purpose of the current study was to evaluate whether similar difference exists in the pharmacokinetics of (S)-warfarin, a prototype substrate of CYP2C9. Validating this systemic reduction through robust *in vivo* pharmacokinetic profiling is critical for safely guiding the use of narrow-therapeutic-index drugs. Furthermore, these findings address the recognized genomic diversity gap, providing essential precision dosing foundations for this cohort while serving as a vital proxy for the underrepresented broader Ethiopian population.

**Methods:**

Single dose of warfarin (20 mg) was administered to 150 Ethiopians and 180 non-Ethiopian healthy, non-smokers subjects. CYP2C9 activity was assessed by (S)-warfarin oral clearance and INR was monitored for 120 h post-dose. CYP2C9 genotyping was performed by direct sequencing.

**Results:**

CYP2C9 genotype correlated with the (S)-warfarin oral clearance in both ethnic groups (p < 0.001). Despite the predominance of CYP2C9*1/*1 genotype in the Ethiopian group, (S)-warfarin oral clearance was reduced by 12.3% as compared to the non-Ethiopian (p < 0.001). Comparison limited to carriers of CYP2C9*1/*1 genotype yielded higher difference of 15.3% (p < 0.001). Stratified VKORC1 genotype analysis revealed significantly greater pharmacodynamic effect in Ethiopians as compared to non-Ethiopians carrying VKORC1 AB and BB haplotypes (AUCINR120: p < 0.04 and p < 0.004 respectively; INRMAX: p < 0.03 and p < 0.002, respectively).

**Conclusion:**

CYP2C9 activity is modestly reduced in Ethiopians as compared to non-Ethiopians thus corroborating our findings regrading phenytoin metabolism. Ethiopian Jews may be susceptible to experience adverse effects when administered CYP2C9 substrate characterized by a narrow therapeutic window. The cause for decreased CYP2C9 activity in Ethiopians is currently under investigation.

## Introduction

CYP2C9, an important member of the cytochrome P450 superfamily, is widely recognized as a clinically actionable pharmacogene (Daly et al., 2018) It accounts for 20% of cytochrome P450 in the liver mediating the oxidative metabolism of approximately 13% of drugs in common use (Zanger and Schwab, 2013). The *in-vivo* activity of CYP2C9 is characterized by marked interindividual variability. Based on research that was predominantly conducted in Caucasians, much of this variability is attributed to common genetic polymorphisms coding for protein with reduced enzymatic activity (Shaul et al., 2017). This is of crucial clinical importance since some of CYP2C9 substrates have a narrow therapeutic window (Daly et al., 2018). For example, the metabolism of (*S*)-warfarin, the more active enantiomer of warfarin, is primarily metabolized by CYP2C9. The use of warfarin poses significant challenges in achieving optimal anticoagulation due to its narrow therapeutic index and the existence of considerable interindividual variability in response. While previous studies have shown that warfarin dose requirements vary widely across ethnic groups (Blann et al., 1999), most clinical guidelines and research have focused on explaining these differences through known allele variants, particularly in *CYP2C9* and *VKORC1* genes (Lee and Klein, 2013).

Despite accounting for over 20% of the global disease burden, African populations remain significantly underrepresented in pharmacogenomic research (Niohuru, 2023; Magavern et al., 2022). Of more than 100 drugs with established clinical pharmacogenomics guidelines, only about 15 have been comprehensively studied in African contexts (Radouani et al., 2020). This disparity is reflected in the poor performance of dosing algorithms, such as those used for warfarin when used in African populations. Ethnicity-based warfarin dosing models, including the widely used IWPC (International Warfarin Pharmacogenetics Consortium) algorithm, demonstrates higher predictive accuracy in individuals of European descent explaining approximately 43% of dose variability compared to only 20%–30% in individuals of African ancestry. This reduced accuracy is largely attributed to the underrepresentation of African-specific genetic variants in model development cohorts (Drozda et al., 2015; Perera et al., 2013; Kimmel et al., 2013; Johnson et al., 2017). Warfarin is still the primary choice for anticoagulation in Africa. Thus, this gap in knowledge is not only of clinical importance but it is especially concerning given Africa’s unparalleled genetic diversity and the opportunity it presents to advance global personalized medicine (Sibomana, 2024).

To address the critical pharmacogenomic gap outlined above, our study harnesses warfarin as a prototype drug and the Ethiopian Jewish community as a clinical model. First and foremost, investigating this cohort addresses an immediate localized clinical need. Numbering more than 177,000 individuals in Israel, the Ethiopian Jewish community possesses a distinct genetic structure that differentiates them from the broader Israeli public and other Jewish diaspora populations (Behar et al., 2010). Yet, their pharmacogenetic characterization particularly regarding the cytochrome P450 enzymes remains critically scarce (Abu Ghosh et al., 2020). Establishing dedicated, cohort-specific baseline data is therefore imperative to ensure the safe and accurate application of genotype-guided clinical decision-making within this specific population. Furthermore, studying this cohort simultaneously provides a broader methodological benefit. Leading genomic initiatives, such the Human Heredity and Health in Africa (H3Africa) Consortium emphasizes that precision medicine requires the establishment of genomic reference data for distinct, local populations (Mulder et al., 2018), because Africa possesses vast, fine-scale regional genetic heterogeneity (Africa et al., 2020). Since Ethiopian Jews share close genetic ties with indigenous East African populations, insights from this cohort serve as a highly valid proxy for broader East African pharmacogenetic variability (Ali et al., 2020; Behar et al., 2010).

We have recently shown that the metabolism of phenytoin, a prototype CYP2C9 substrate, is significantly reduced in Ethiopian Jews (EJ) compared to Non-Ethiopian Jews (NEJ) not carrying the most common allelic variants in Caucasians (i.e., CYP2C9*2 and CYP2C9*3) and the “African” variant alleles (i.e., CYP2C9*5, CYP2C9*6, CYP2C9*8 and CYP2C9*11) (Abu Ghosh et al., 2020). In the current study, we aim to compare the metabolism and pharmacodynamic effect of (*S*)-warfarin, a different substrate of CYP2C9, between Ethiopian and non-Ethiopians Jews residing in Israel. By doing so, we seek to assess whether the decrease in phenytoin metabolism observed in Ethiopian Jews extends beyond a single drug. This would help to either reinforce or revise our understanding of CYP2C9 activity in this population and determine whether these differences are substrate-specific or indicative of a broader phenomenon of pharmacokinetic variability.

Valid comparison of warfarin pharmacokinetics and pharmacodynamics between two populations of different ethnic background cannot be performed without taking into consideration the presence of known genetic markers that might have different impact in these populations. Specifically, our study included the genetic analysis of rs12777823G>A which has been shown in multiple studies to be associated with reduced CYP2C9 activity and specifically S-warfarin clearance only among Africans (Perera et al., 2013; Drozda et al., 2015). Similarly, VKORC1 genetic analysis, the key genetic determinant of warfarin pharmacodynamics, was also included in the study.

## Materials and methods

### Subjects

In our previous study aimed to evaluate the relative decrease in (*S*)-warfarin clearance in carriers of different *CYP2C9* genotypes, mean (*S*)-warfarin oral clearance was 209 ± 86 mL/h^3^. Thus, detection of 15% difference in (*S*)-warfarin oral clearance between unequal sizes of Ethiopian and non-Ethiopian Jews with a power of 90%, 5% α (type I error) and 0.05 level of significance requires enrollment of 170 subjects in one group (non-Ethiopians) and 141 subjects in the second group (Ethiopians). Thus, the original study cohort which compared phenytoin metabolic ratio between 150 Ethiopian and 150 non-Ethiopian Jews (Abu Ghosh et al., 2020) residing in Israel was enriched by additional 30 non-Ethiopian Jews. Study participants were healthy based on self-statement and unremarkable findings in physical examination, non-smokers, age 18–50 years and not treated regularly by any medication including oral contraceptives. Potential candidates were considered as “Ethiopians” if both parents and grandparents were born in Ethiopia prior to immigration to Israel. No matching in demographic characteristics was made between the Ethiopian and the non-Ethiopian study groups. The study protocol was approved by the Hadassah University Medical Center Institutional Review Board and following a detailed explanation regarding the characteristics of the study, all subjects gave their written consent to participate in the study (Clinicaltrials.gov NCT00162474).

### CYP2C9 phenotyping

CYP2C9 activity was assessed in all subjects using phenytoin as described elsewhere (Abu Ghosh et al., 2020; Caraco et al., 2001; Shaul et al., 2022). The comparison between Ethiopian and non-Ethiopian Jews among 300 subjects (150 in each ethnic group) was previously published (Abu Ghosh et al., 2020). Briefly, following 8 h of fasting, a single dose of 300 mg phenytoin was administered together with 250 mL of water. Fasting was continued for additional 4 h and urine was collected over 24 h in two separate intervals, 0–12 h and 12–24 h. Blood sample was obtained 12 and 24-h following phenytoin administration. Phenytoin Metabolic Ratio (PMR), a validated marker of CYP2C9 activity *in-vivo* was derived from the ratio of p-HPPH content in 24 urine collection to plasma phenytoin concentration 12 (i.e., PMR24/12) or 24 (i.e., PMR24/24) hours following phenytoin administration.

### Warfarin pharmacokinetics and pharmacodynamics

At least 1 week after phenytoin intake and following 8 h of overnight fast, a single dose of warfarin was administered together with 250 mL of water. All subjects received 20 mg of warfarin except for carriers of 2 variant alleles (i.e., CYP2C9*2/*2, CYP2C9*2/*3 and CYP2C9*3/*3) who received only 10 mg. Fasting was continued for additional 4 h when a standardized meal was provided. Periodic blood samples were obtained prior to and 1, 2, 3, 4, 6, 8, 10, 12, 14, 24, 30, 36, 48, 54, 60, 72, 96 and 120-h after warfarin intake. The plasma concentration of (*S*)- and (*R*)-warfarin was determined using an enantioselective high-performance liquid chromatography as published previously (Wanounou et al., 2022). The oral clearance of (*S*)- and (*R*)-warfarin were calculated from the ratio of warfarin dose (half of the racemic dose for each enantiomer) divided by the area under the plasma concentration time curve of the respective enantiomer extrapolated to infinity.

The pharmacodynamics of warfarin was evaluated by periodic measurement of the International Normalized Ratio (INR), prior to and 12, 24, 36, 48, 60, 72, 96 and 120-h following warfarin administration. The extent of anticoagulation over the entire study period was evaluated by calculating the area under the INR curve from zero to 120 h (AUCINR_120_). The highest INR value during the entire study period (INR_MAX_) was determined by direct observation.

### Genetic analysis

Genomic DNA was extracted from peripheral leukocytes using traditional phenol-chloroform extraction procedure. CYP2C9 genetic analysis was performed by direct sequencing of all 9 exons with surrounding intronic boundaries (The Center for Genomic Technologies, The Hebrew University of Jerusalem, Givat Ram).

The identification of rs12777823 C>A at the promoter region of the CYP2C cluster gene was performed by direct sequencing of the relevant region. *VKORC1* haplotype group combinations and rs61742245 C>A (D36Y) genetic analysis were done by identification of 16:31093557C>T (6484C>T) and 16:31094624C>A polymorphisms through direct sequencing of the relevant regions on chromosome 16 (Rieder et al., 2005; Kurnik et al., 2012).

### Data analysis

Demographic details and the distribution of *CYP2C9* genotypes are presented for each ethnic group separately (Table 1). Based on our previous findings regarding phenytoin metabolism, comparison of demographic details, warfarin pharmacokinetics and pharmacodynamics was done twice, once for the entire cohort irrespective of *CYP2C9* genotype and again only for carriers of CYP2C9*1/*1 genotype. The distribution of CYP2C9 genotypes among Ethiopians and non-Ethiopians was compared using the chi-square test. Differences in demographics (i.e., age, weight, BMI), warfarin pharmacokinetics and pharmacodynamics between the study groups were performed using unpaired t-test or the non-parametric Mann-Whitney U test as appropriate. The impact of demographic details (i.e., age, gender, weight) and genetic analysis on warfarin enantioselective pharmacokinetics and pharmacodynamics were evaluated through univariate analysis using Pearson correlation test. Variables that correlated with either warfarin pharmacokinetics or pharmacodynamics with a p-value of less than 0.1 were entered into multiple regression analysis model using the stepwise approach. Statistical analysis was performed using the SPSS software package (IBM SPSS Statistics, version 26, Chicago IL, USA). Data are presented as mean ± SD and p value of less than 0.05 was considered to denote statistical significance. Results were not corrected for multiple testing.

**TABLE 1:** Demographic details, *CYP2C9*, rs12777823, *VKORC1* and rs61742245 genotypes in Ethiopian and non-Ethiopian Jews.

Variable	​	Ethiopians (N = 150) Mean ± SD	Non-Ethiopians (N = 180) Mean ± SD	p Value
Demographics				
​	Age	24.95 ± 5.44	24.61 ± 5.52	NS
​	Weight	62.16 ± 12.21	68.59 ± 12.54	<0.001
​	BMI	22.36 ± 3.70	23.91 ± 3.48	<0.001
​	BSA	1.579 ± 0.435	1.625 ± 0.490	<0.003
​	Male/Female	66/84	83/97	NS
CYP2C9 genotype				
​	*1/*1	118	93	​
​	*1/*2	4	44	​
​	*1/*3	2	30	​
​	*1/*9	5	0	​
​	*1/*11	13	0	​
​	*1/*14	0	1	​
​	*1/*58	1	0	​
​	*1/*73	1	0	​
​	*2/*2	0	1	​
​	*2/*3	0	8	​
​	*2/*9	1	0	​
​	*2/*11	3	0	​
​	*3/*3	0	1	​
​	*3/*8	0	1	​
​	*3/*42	0	1	​
​	*9/*73	1	0	​
​	F126C and G416D	1	0	​
​	​	​	​	<0.001
rs12777823G>A				
​	GG	105	143	​
​	GA	39	35	​
​	AA	6	2	​
​	​	​	​	<0.07
VKORC1 genotype				
​	AA	17	43	​
​	AB	75	89	​
​	BB	58	48	​
​	​	​	​	<0.005
rs61742245C>A				
(D36Y)	CC	104	171	​
​	CA	44	9	​
​	AA	2	0	​
​	​	​	​	<0.001

## Results

All 330 subjects completed the study. Subject’s demographics and CYP2C9 genetic analysis are presented in Table 1. No significant difference was noted in age and gender distribution between the Ethiopian and the non-Ethiopian study groups (p > 0.5). However, Weight, BMI and BSA were significantly smaller by 9.4%, 6.5% and 2.8%, respectively among the Ethiopians (p < 0.001, p < 0.001 and p < 0.003, respectively).

### Genetic analysis

#### *CYP2C9* genetic analysis

The sequence of all nine CYP2C9 exons identified 10 subjects in the Ethiopian group as carriers of rare novel variant alleles (CYP2C9*9, CYP2C9*58, CYP2C9*73 and combination of F126C with G416D) other than the Caucasian (CYP2C9*2, CYP2C9*3) or African (CYP2C9*5, CYP2C9*6, CYP2C9*8 and CYP2C9*11) alleles. Among the non-Ethiopian study group, 3 subjects were identified as carriers of rare novel variant alleles (CYP2C9*8, CYP2C9*14 and CYP2C9*42). The fraction of subjects carrying the wild-type (i.e., CYP2C9*1/*1) genotype was significantly greater in the Ethiopian as compared with the non-Ethiopian group accounting for 78.7% and 51.7% of the entire cohort, respectively (p < 0.001).

The distribution of rs12777823 genotypes among Ethiopian and non-Ethiopian Jews is shown in Table 1. The frequency of variant allele A carriers was significantly greater among the Ethiopian group (30.0% vs. 20.6%, respectively, p < 0.05).

#### VKORC1 and D36Y genetic analysis

The distribution of VKORC1 haplotype group combinations and rs61742245 C>A (D36Y) genotypes is presented in Table 1. The distribution of VKORC1 genotypes exhibited significant interethnic difference (p < 0.005). The frequency of the more sensitive *VKORC1 AA* genotype was 2-fold lower in Ethiopians (11.3% vs. 23.9%, respectively, p < 0.005) with the opposite trend for the resistant *VKORC1 BB* genotype (38.7% vs. 26.7%, respectively, p < 0.02). The corresponding figures for the cohort consisting of only CYP2C9*1/*1 genotype were similar (VKORC1 AA: 11.9% vs. 30.1%, respectively, p < 0.001; VKORC1 BB: 39.0% vs. 21.5%, respectively, p < 0.007). The distribution of VKORC1 D36Y variant was significantly different between Ethiopians and non-Ethiopians (p < 0.001). Among Ethiopians, the minor allele frequency of D36Y polymorphism was 16.0% and significantly greater than frequency of 2.5% noted among non-Ethiopians (p < 0.001). The corresponding frequency in the cohort consisting of only CYP2C9*1/*1 genotype was similar (14.4% vs. 2.2%, respectively, p < 0.001).

### Warfarin enantioselective pharmacokinetics

#### (*S*)-warfarin

**FIGURE 1:**
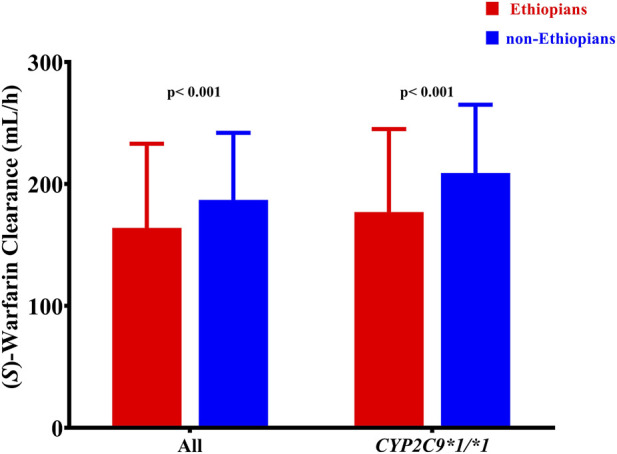
The oral clearance of (*S*)-warfarin in Ethiopian (red bars) and non-Ethiopian (blue bars) Jews. Comparison is shown for the entire cohort irrespective of *CYP2C9* genotype (ALL) and among carriers of CYP2C9*1/*1 genotype.

*CYP2C9* genotype correlated with the oral clearance of (*S*)-warfarin in both study ethnic groups (Kruskal–Wallis, p < 0.001). Despite the predominance of CYP2C9*1/*1 genotype in the Ethiopian group, the mean oral clearance of (*S*)-warfarin was reduced by 12.3% as compared to the non-Ethiopian (p < 0.001) (Table 2; Figure 1). When the comparison between the Ethiopian and the non-Ethiopian groups was made only among carriers of CYP2C9*1/*1 genotype the decrease in (*S*)-warfarin oral clearance rose up to 15.3% (p < 0.001) (Table 2; Figure 1).

**TABLE 2:** (*S*)- and (*R*)-warfarin oral clearance among Ethiopians and non-Ethiopians among carriers of different *CYP2C9* and rs12777823 genotypes.

Enantiomer	Genotype	Ethiopians (N)	Non-Ethiopians (N)	p Value
(*S*)-Warfarin CL (mL/hr)	*CYP2C9* genotype	​	​	​
​	All *CYP2C9* genotypes	164 ± 69 (150)	187 ± 55 (180)	<0.001
​	CYP2C9*1/*1	177 ± 68 (118)	209 ± 56 (93)	<0.001
​	All *CYP2C9* genotypes	​	​	​
​	rs12777823G>A	​	​	​
​	GG	173 ± 71 (105)	185 ± 48 (143)	<0.03
​	GA & AA	142 ± 59 (45)	195 ± 76 (37)	<0.001
​	CYP2C9*1/*1	​	​	​
​	rs12777823G>A	​	​	​
​	GG	178 ± 72 (92)	209 ± 43 (65)	<0.001
​	GA & AA	174 ± 53 (26)	211 ± 78 (28)	<0.03
(*R*)-Warfarin CL (mL/hr)	​	​	​	​
​	All *CYP2C9* genotypes	111 ± 45 (150)	135 ± 49 (180)	<0.001
​	CYP2C9*1/*1	114 ± 46 (118)	123 ± 37 (93)	>0.1

**FIGURE 2:**
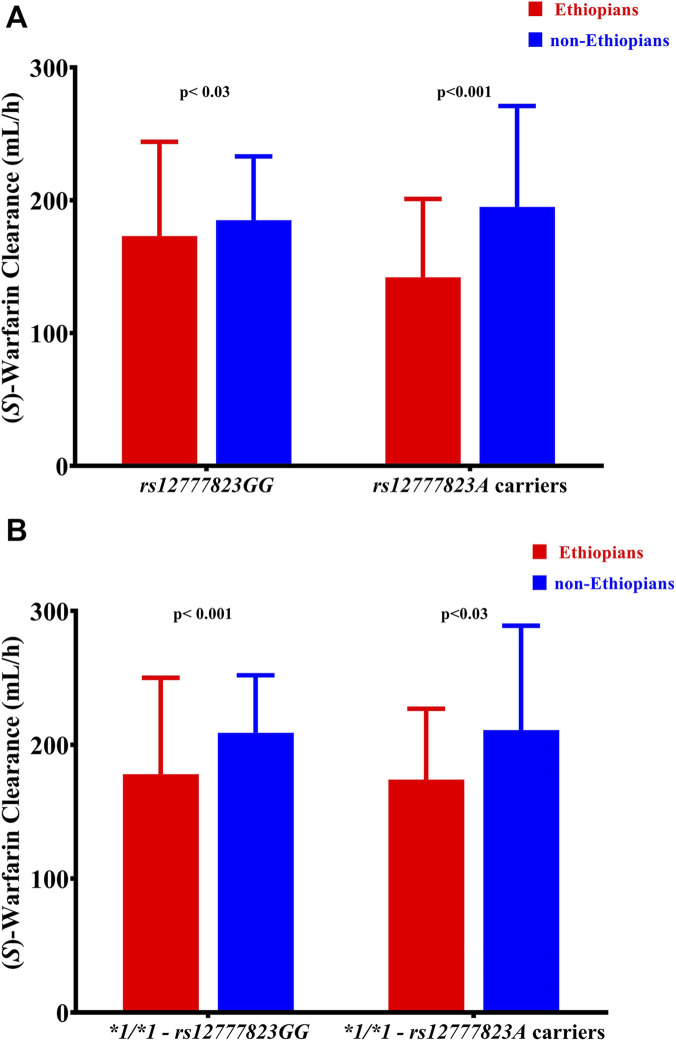
The oral clearance of (*S*)-warfarin in Ethiopian (red bars) and non-Ethiopian (blue bars) Jews carrying different *rs12777823* genotype. **(A)** Comparison is shown for the entire cohort irrespective of *CYP2C9* genotype. **(B)** Comparison is shown only among carriers of CYP2C9*1/*1 genotype.

The oral clearance of (*S*)-warfarin was significantly correlated with rs12777823 genotype among the Ethiopian but not among the non-Ethiopian group (Kruskal–Wallis, p < 0.002, p > 0.5, respectively). The difference in (*S*)-warfarin oral clearance between Ethiopians and non-Ethiopians was particularly prominent among carriers of the variant A allele but only subtle among non-carriers (p < 0.001, p < 0.03, respectively) (Table 2; Figure 2A). Thus, when the entire cohort was considered irrespective of *CYP2C9* genotype, much of the difference between Ethiopian and non-Ethiopians in the oral clearance of (*S*)-warfarin could be possibly attributed to higher frequency of variant allele A among Ethiopians and the interethnic differential impact of this polymorphism on (*S*)-warfarin clearance. However, analysis restricted to carriers of CYP2C9*1/*1 revealed significant difference in (*S*)-warfarin oral clearance between Ethiopians and non-Ethiopians among both carriers and non-carriers of the variant A allele (p < 0.03, p < 0.001, respectively) (Table 2; Figure 2B). The oral clearance of (*S*)-warfarin was significantly correlated with *CYP2C9* and rs12777823G>A genotypes as well as several host factors including age, weight and BSA. These variables were entered into multiple regression model using the stepwise approach. The final model could explain 30.9% of the variance in (*S*)-warfarin oral clearance (p < 0.001) consisting of weight (11.9%), CYP2C9*1/*1 genotype (11.3%), carriage of CYP2C9*2 allele (3.9%) Ethnicity (2.7%) and carriage of CYP2C9*11 allele (1.1%).

#### (*R*)-warfarin

*CYP2C9* genotype was associated with (R)-warfarin oral clearance among non-Ethiopians (p < 0.001) but not among Ethiopians (p > 0.2). Mean oral clearance of (*R*)-warfarin was reduced by 17.7% among Ethiopian as compared to non-Ethiopian Jews (111 ± 45 vs. 135 ± 49 mL/h, respectively, p < 0.001) (Table 2; Supplementary Figure S1). No significant interethnic difference in (*R*)-warfarin oral clearance was noted when the comparison was made among carriers of CYP2C9*1/*1 genotype (Table 2, Supplementary Table S1).

### Pharmacodynamics

AUCINR_120_ and INR_MAX_ were not associated with *CYP2C9* genotype in either the Ethiopian (p > 0.4) or the non-Ethiopian group (p > 0.2). However, AUCINR_120_ and INR_MAX_ varied significantly among carriers of different *VKORC1* genotypes in both Ethiopians and non-Ethiopians (p < 0.001) (Supplementary Table S1). Similar results were obtained for both Ethiopians and non-Ethiopians when the analysis was restricted to carriers of CYP2C9*1/*1 genotype (p < 0.001, Supplementary Table S2).

Among the Ethiopian group, AUCINR_120_ but not INR_MAX_ was correlated with rs61742245 C>A polymorphism (Kruskal–Wallis p < 0.05) so that it was significantly greater in CC as compared to CA carriers. Among the non-Ethiopians neither AUCINR_120_ nor INR_MAX_ were associated with rs61742245 C>A polymorphism (p > 0.2) (Supplementary Table S1). Analysis limited to carriers of CYP2C9*1/*1 revealed significant differences in both AUCINR_120_ and INR_MAX_ among carriers of different rs61742245 C>A genotypes in the Ethiopian (Kruskal–Wallis p < 0.006 and p < 0.04, respectively) but not in the non-Ethiopian group (Supplementary Table S2).

**FIGURE 3:**
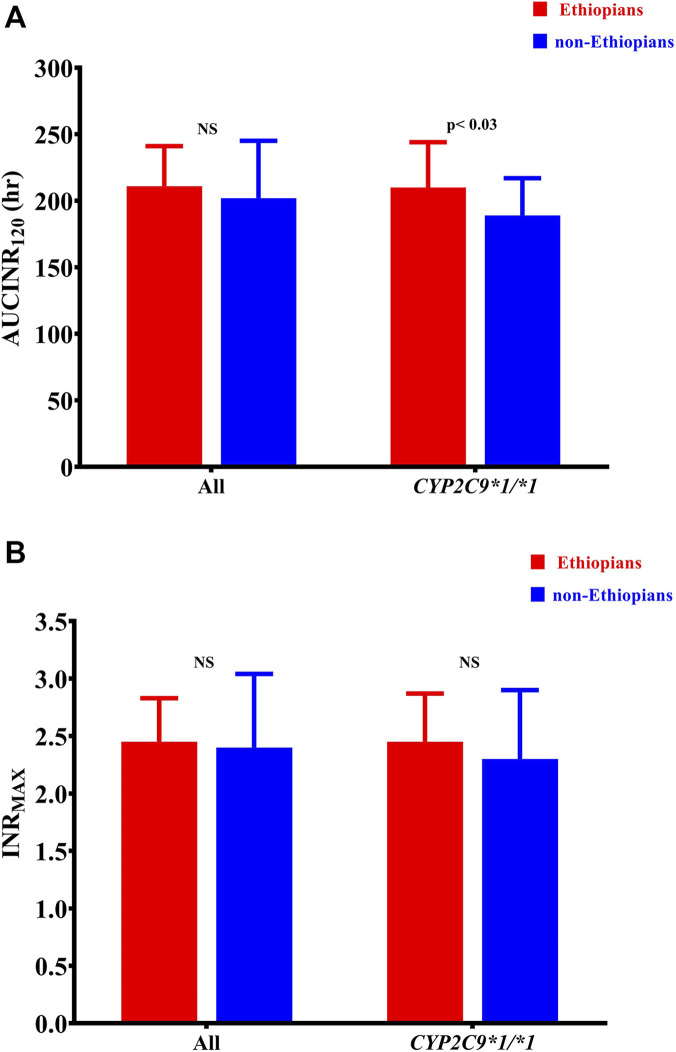
Area under the INR curve from 0 to 120 hours (AUCINR120) **(A)** and maximal observed INR (INRMAX) **(B)** in Ethiopian (red bars) and non-Ethiopian (blue bars) Jewish carriers of the VKORC1 AA genotype. Comparisons are shown for the entire cohort irrespective of *CYP2C9* genotype (ALL), and specifically for CYP2C9*1/*1 carriers.

**FIGURE 4:**
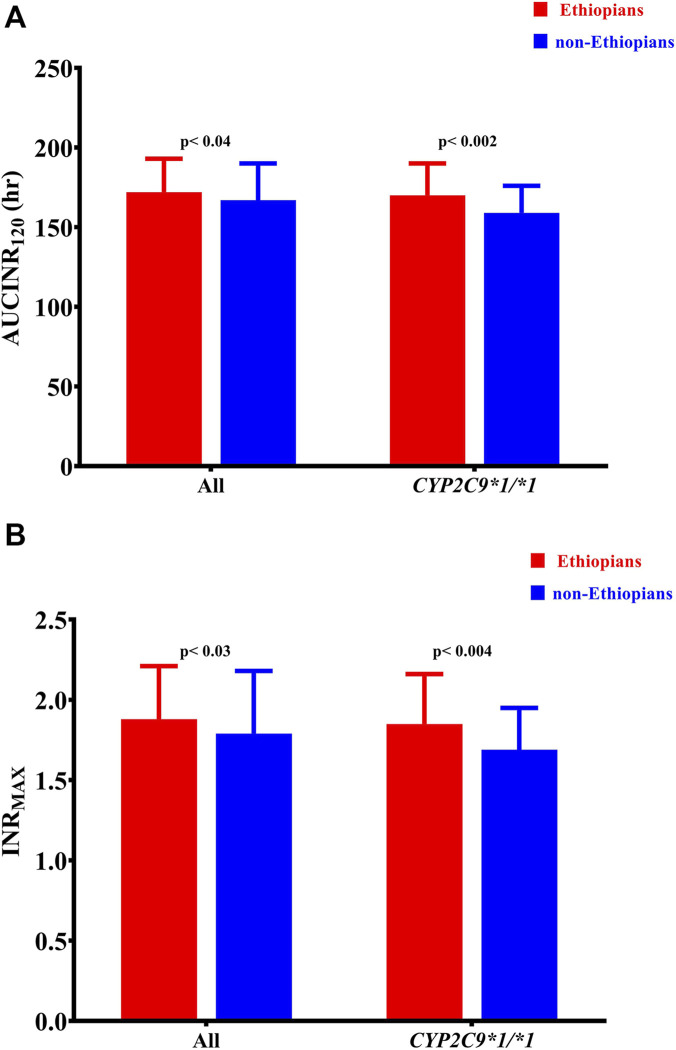
Area under the INR curve from 0 to 120 hours (AUCINR120) **(A)** and maximal observed INR (INRMAX) **(B)** in Ethiopian (red bars) and non-Ethiopian (blue bars) Jewish carriers of the VKORC1 AB genotype. Comparisons are shown for the entire cohort irrespective of *CYP2C9* genotype (ALL), and specifically for CYP2C9*1/*1 carriers.

**FIGURE 5:**
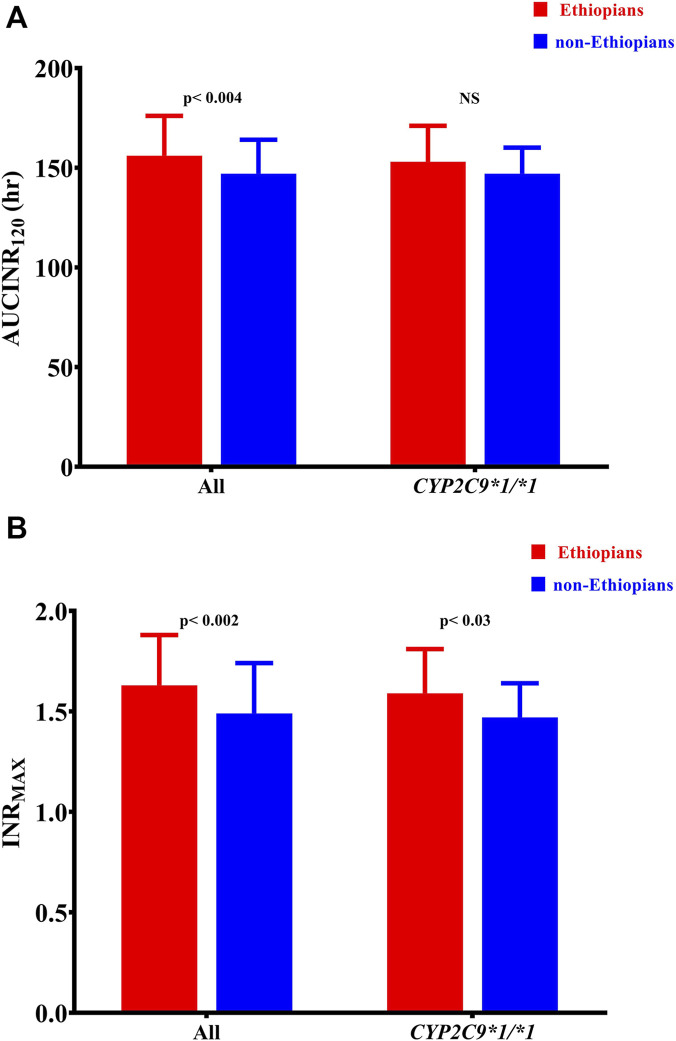
Area under the INR curve from 0 to 120 hours (AUCINR120) **(A)** and maximal observed INR (INRMAX) **(B)** in Ethiopian (red bars) and non-Ethiopian (blue bars) Jewish carriers of the VKORC1 BB genotype. Comparisons are shown for the entire cohort irrespective of *CYP2C9* genotype (ALL), and specifically for CYP2C9*1/*1 carriers.

No significant differences were noted between the Ethiopian and the non-Ethiopian group in either AUCINR_120_ or INR_MAX_ (Supplementary Table S1; Supplementary Figures S2, S3). Similar results were obtained when the analysis was limited to carriers of CYP2C9*1/*1 genotype (Supplementary Table S2; Supplementary Figures S2, S3). In view of the different distribution of *VKORC1* haplotypes between Ethiopians and non-Ethiopians and the correlation found between *VKORC1* genotype and pharmacodynamics we further evaluated possible interethnic differences in pharmacodynamics using *VKORC1* genotype stratified analysis. Analysis of the entire cohort irrespective of *CYP2C9* genotype revealed a significantly greater AUCINR_120_ and INR_MAX_ among the Ethiopians as compared to non-Ethiopians who were carriers of *VKORC1 AB* and *BB* genotypes (Supplementary Table S1; Figures 3–5) AUCINR_120_; p < 0.04 and p < 0.004, respectively, INR_MAX_; p < 0.03 and p < 0.002, respectively). Interethnic comparison limited to carriers of CYP2C9*1/*1 genotype yielded similar qualitative findings (Supplementary Table S2; Figures 3–5). Thus, AUCINR_120_ was significantly greater in Ethiopians as compared to non-Ethiopians in carriers of *VKORC1 AA* and *AB* genotypes (p < 0.03 and p < 0.002, respectively), whereas INR_MAX_ was significantly higher in Ethiopians as compared to non-Ethiopians among carriers of *AB* and *BB VKORC1* genotypes (p < 0.004 and p < 0.03, respectively).

No significant differences were noted in pharmacodynamic parameters between Ethiopians and non-Ethiopians when comparison was made based on stratification according to D36Y genetic polymorphism (Supplementary Tables S1, S2).

## Discussion

The oral clearance of (*S*)-warfarin, a prototype of CYP2C9 substrate is reduced by 12.3% among Ethiopian as compared to non-Ethiopian Jews and this difference is 15.3% when the comparison was limited to carriers of the wild-type genotype (i.e., CYP2C9*1/*1). The findings in the present study are in line with the results of our previous study indicating 30% mean decrease in phenytoin metabolism, another substrate of CYP2C9, in Ethiopian compared to non-Ethiopian Jews carriers of CYP2C9*1/*1 genotype (Abu Ghosh et al., 2020). Combinedly these findings suggest that CYP2C9 activity is lower in Ethiopian as compared to non-Ethiopian Jews and this difference may exist for additional CYP2C9 substrates other than phenytoin and (*S*)-warfarin.

Interethnic variability in CYP2C9 activity has been previously reported. Hatta et al. have found significant decrease in CYP2C9 activity as evaluated by the urinary ratio of losartan to E3174 in Swedish as compared to Korean subjects carrying the same *CYP2C9* genotype (Hatta et al., 2015). However, in this study *CYP2C9* genotype was determined based on the analysis for only 3 variant alleles (CYP2C9*2, CYP2C9*3 and CYP2C9*13) and therefore the existence of other *CYP2C9* allelic variant that were not tested for, cannot be excluded. In another study, the urinary ratio of diclofenac to 3'-, 4′- and 5′-hydroxy-diclofenc, a marker of CYP2C9 activity, was significantly greater among Cuban-Mestizos as compared to Cuban-White and Spaniards, all carrying the same *CYP2C9* genotype (Lierena et al., 2015). Finally, our findings are in agreement with another study that compared the oral clearance of (*S*)-warfarin between Asian (N = 196), Caucasian (N = 122) and a small group (N = 60) of African American patients on chronic warfarin maintenance treatment (Kubo et al., 2017; Ohara et al., 2019). Using population pharmacokinetic analysis, the authors were able to show a 30% decrease in median oral clearance of (*S*)-warfarin in the African American group as compared to Asians and Caucasians and this difference was present when analysis was done only for carriers of CYP2C9*1/*1 genotype.

Despite the apparent decrease in (*S*)-warfarin oral clearance among the Ethiopian Jews this difference was not translated into greater anticoagulant effect. However, the extent of anticoagulation following a single dose of warfarin was greater in the Ethiopian as compared to the non-Ethiopian group when analysis was stratified by VKORC1 haplotype. Yet, the higher proportion of carriers of the resistant VKORC1 BB genotype among Ethiopians offsets the decrease in clearance of (*S*)-warfarin when the entire Ethiopian and non-Ethiopians cohorts were considered.

The strength of this study lies in its design, which enable comparison of the pharmacokinetics and pharmacodynamics of warfarin between 2 ethnic cohorts consisting of healthy, non-smokers subjects with similar age and proportion of males and females. Thus, the confounding effect of known determinants of (*S*)-warfarin clearance such as age, gender, drug intake and disease states can be excluded.

Our study has several limitations. First, since matching between the Ethiopian and the non-Ethiopian group was not done, weight, BMI and BSA were modestly smaller by 9.4%, 6.5% and 2.8%, respectively in the Ethiopian group. Weight or Body Surface Area (BSA) are often included in many but not all warfarin dosing algorithms (Nagai et al., 2015; Cross et al., 2024). However, the contribution of weight or BSA on (*S*)-warfarin clearance is considerably small compared to other determinants. For example, decrease in weight by 10 kg from 93 kg. to 83 kg. or in BSA from 1.99 m^2^ to 1.89 m^2^ is associated with only 5% reduction in (*S*)-warfarin clearance (Nagai et al., 2015). In the present study the difference in weight between Ethiopian and non-Ethiopian groups was 6.6 kg and 0.04 m^2^ in BSA. Thus, it is unlikely that the interethnic difference in the oral clearance of (*S*)-warfarin noted in the present study is attributed to variability in weight or BSA. Second, our results were obtained following administration of a single relatively large warfarin dose. It is unclear if similar findings will be obtained following repeated intake of the customary clinical dose of warfarin. However, prediction of (*S*)-warfarin pharmacokinetics is most helpful clinically during the initial phase of treatment when the individual’s response to warfarin is unknown. Third, diet composition has been shown to modulate the activity of cytochrome P450 enzymes including CYP2C9 (Hidaka et al., 2008; Kimura et al., 2010; Wang et al., 2020). In the present study diet consumed by the participants was not controlled for and therefore the possibility that regular consumption of spices or herbs which are part of the traditional Ethiopian diet might inhibit the activity of CYP2C9 cannot be ruled out.

The cause for the decreased activity of CYP2C9 in Ethiopian Jews is currently not known. The possibility that multiple yet rare coding region polymorphisms may exist in the Ethiopian group has been practically ruled out since CYP2C9*1/*1 genotype was determined by complete sequence of all 9 exons. The rs12777823G>A polymorphism in the CYP2C cluster is associated with reduced warfarin dose requirement among African Americans but not among Caucasians (Perera et al., 2013). Furthermore, the findings of a small pharmacokinetic study among African Americans suggests that carriers of rs12777823 A variant allele(s) exhibit reduced (*S*)-warfarin oral clearance (Perera et al., 2013). However, analysis stratified by rs12777823G>A polymorphism demonstrated significant decrease in (*S*)-warfarin oral clearance in the Ethiopian group irrespective of variant A allele carrier status. These findings suggest that the interethnic variability in (*S*)-warfarin oral clearance cannot be accounted for by rs12777823G>A genetic polymorphism. Finally host and environmental factors could influence difference in CYP2C9 activity. For example, increased age has been associated with higher sensitivity to warfarin which is at least partly accounted for by decreased warfarin clearance (Jensen et al., 2012). Using population pharmacokinetic approach, increase in age by 10 years was estimated to be associated with 5% decrease in (*S*)-warfarin clearance (Kubo et al., 2017). In some studies gender was found to modulate CYP2C9 activity so that female patients were estimated to exhibit 10% decrease in (*S*)-warfarin clearance (Kubo et al., 2017; Ohara et al., 2019). There is a long list of drugs capable of altering CYP2C9 activity through either inhibition or induction of enzyme activity (Daly et al., 2018). However, none of the aforementioned host or environmental factors could account for the decrease in (*S*)-warfarin oral clearance among Ethiopian Jews since participants were healthy non-smokers and not treated regularly by any medication with almost identical mean age and similar proportion of males and females. Proteomic analysis conducted in liver samples from Black and White Americans suggests that CYP2C9 protein expression is significantly lower in the former ethnic group. Stratified analysis by *CYP2C9* genotype indicates that the racial difference was particularly prominent in carriers of CYP2C9*8 and CYP2C9*11 whereas the difference in carriers of CYP2C9*1/*1 genotype was marginal and did not reach statistical significance (Jung et al., 2025). Thus, the difference in CYP2C9 activity between Ethiopian and non-Ethiopian Jews could be mediated by differences in translational regulation, epigenetic modifications, or other non-coding regulatory elements that play a crucial role in modulating CYP2C9 expression and activity (Fekete et al., 2021; Montalvo et al., 2025). This study underscores the importance of investigating non-coding regulatory factors in pharmacogenomics, particularly in populations that have been historically underrepresented in genetic research.

The 15.3% decrease in (*S*)-warfarin oral clearance observed in Ethiopian compared to non-Ethiopian Jews (both CYP2C9*1/1) is similar to the reduction reported in the literature for individuals heterozygous for CYP2C9*2 as compared to carriers of wild-type genotype (Shaul et al., 2017). Notably, CPIC guidelines classify CYP2C9*1/*2 carriers as having a decreased function allele (average activity score of 0.5), defining them as intermediate metabolizers (Theken et al., 2020). This classification is associated with an increased risk of adverse events with NSAID treatment and necessitates dose adjustments for drugs such as acenocoumarol. Our findings raise an important question: should Ethiopian ancestry be considered equivalent to carrying a decreased function allele, warranting similar pharmacogenomic considerations and dose adjustments? Further research is needed to determine whether Ethiopian Jews, despite being genotypically wild-type, require individualized dosing strategies similar to those recommended for CYP2C9 intermediate metabolizers.

While population-based studies have established that warfarin dose requirements in African Americans are generally higher than in Caucasians (Blann et al., 1999), findings in previous studies of reduced (*S*)-warfarin clearance in African Americans (Kubo et al., 2017) presents an apparent contradiction. This discrepancy can be challenging to reconcile; however, it may be explained by the greater-than-expected influence of pharmacodynamic (PD) factors, particularly variations in VKORC1, the target enzyme of warfarin. The higher prevalence of *VKORC1* variants associated with warfarin resistance in this population counterbalance the impact of reduced CYP2C9-mediated clearance, ultimately leading to higher dose requirements despite lower rate of warfarin metabolism. Notably, in a sub-analysis comparing individuals with identical *VKORC1* genotypes, the observed pharmacokinetic differences were translated into pharmacodynamic differences, as reflected by higher AUCINR_120_ and INR_MAX_. This finding reinforces the notion that interethnic variability in warfarin response is shaped by a complex interplay between pharmacokinetic and pharmacodynamic factors, highlighting the importance of personalized dosing strategies.

## Conclusion

This study demonstrates significant interethnic differences in both the pharmacokinetics and anticoagulation response of (S)-warfarin between Ethiopian and non-Ethiopian Jews. The findings in the present study suggest that *in-vivo* CYP2C9 activity is reduced in Ethiopian Jews, potentially increasing the risk for adverse drug reactions when narrow therapeutic window drugs such as (*S*)-warfarin are prescribed. These results corroborate our earlier findings with phenytoin and underscore the need for tailored dosing strategies in different ethnic populations.

### Previous presentations

A part of this work was presented in a poster at the 2023 ASCPT annual meeting.

## Data Availability

The data presented in the study are deposited in the Figshare repository (https://figshare.com/s/12cdc10f85e63f9b4baf).
