# Supplementary material for: Comparison of CYP2C9 activity between Ethiopian and non-Ethiopian Jews: an interethnic study of (S)-warfarin pharmacokinetic and pharmacodynamic
Source: Front Pharmacol. 2026 Jun 23;17:1836874. doi: 10.3389/fphar.2026.1836874 (PMC13337887; doi:10.3389/fphar.2026.1836874)
Supplement: Supplementary file 1 [file Table1.docx]

Table S1: Comparison of AUCINR_120_ and INR_MAX_ between Ethiopians and non-Ethiopians in carriers of different *VKORC1* and D36Y genotypes.

|  |  | Ethiopians  (N) | Non-Ethiopians  (N) | p value |
| --- | --- | --- | --- | --- |
| AUCINR_120_ (hours) |  |  |  |  |
|  | All *CYP2C9* genotypes | 171 ± 27  (150) | 170 ± 34  (180) | NS |
|  |  |  |  |  |
|  | *VKORC1* Genotype |  |  |  |
|  | AA | 211 ± 30  (17) | 202 ± 43  (43) | NS |
|  | AB | 172 ± 21  (75) | 167 ± 23  (89) | < 0.04 |
|  | BB | 156 ± 20  (58) | 147 ± 17  (48) | < 0.004 |
|  |  |  |  |  |
|  | rs61742245C>A |  |  |  |
|  | CC | 173 ± 28  (N=104) | 171 ± 34  (N=171) | NS |
|  | CA | 163 ± 26  (44) | 156 ± 17  (9) | NS |
|  | AA | 182 ± 2  (2) | (0) |  |
|  |  |  |  |  |
| INR_MAX_ |  |  |  |  |
|  | All CYP2C9 genotypes | 1.85 ± 0.39  (150) | 1.86 ± 0.55  (180) | NS |
|  |  |  |  |  |
|  | *VKORC1* Genotype |  |  |  |
|  | AA | 2.45 ± 0.38  (17) | 2.40 ± 0.64  (43) | NS |
|  | AB | 1.88 ± 0.33  (75) | 1.79 ± 0.39  (89) | < 0.03 |
|  | BB | 1.63 ± 0.25  (58) | 1.49 ± 0.25  (48) | < 0.002 |
|  |  |  |  |  |
|  | rs61742245C>A |  |  |  |
|  | CC | 1.88 ± 0.39  (104) | 1.87 ± 0.55  (171) | NS |
|  | CA | 1.76 ± 0.38  (44) | 1.62 ± 0.23  (9) | NS |
|  | AA | 1.99 ± 0.13  (2) | (0) |  |
