# Supplementary figures and images for: Comparison of CYP2C9 activity between Ethiopian and non-Ethiopian Jews: an interethnic study of (S)-warfarin pharmacokinetic and pharmacodynamic
Source: Front Pharmacol. 2026 Jun 23;17:1836874. doi: 10.3389/fphar.2026.1836874 (PMC13337887; doi:10.3389/fphar.2026.1836874)

**Fig. S2: AUCINR<sub>120</sub> in Ethiopians and non-Ethiopians**

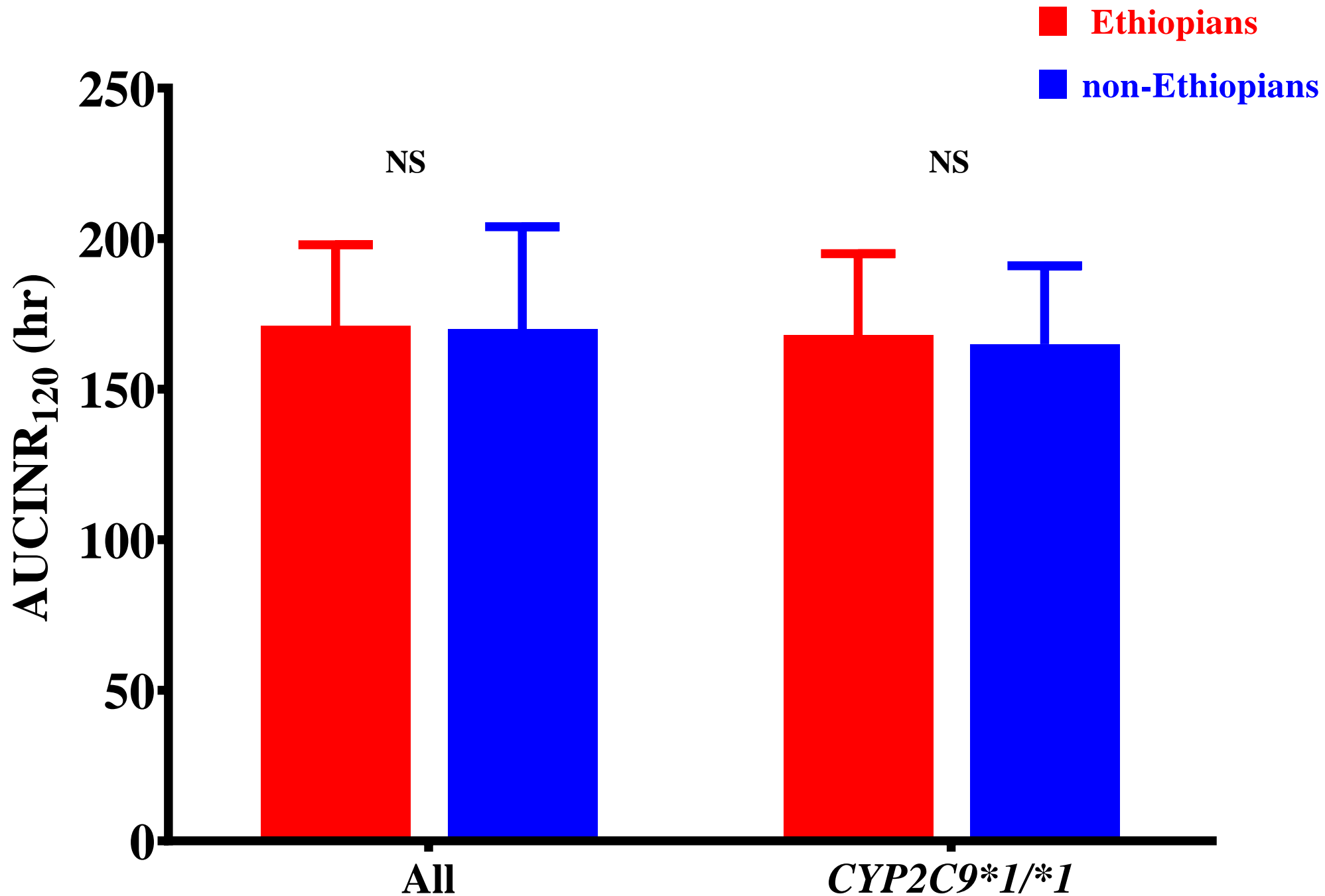

Supplement: Supplementary file 2 [file Image2.pdf]

**Fig. S3: INR<sub>MAX</sub> in Ethiopians and non-Ethiopians**

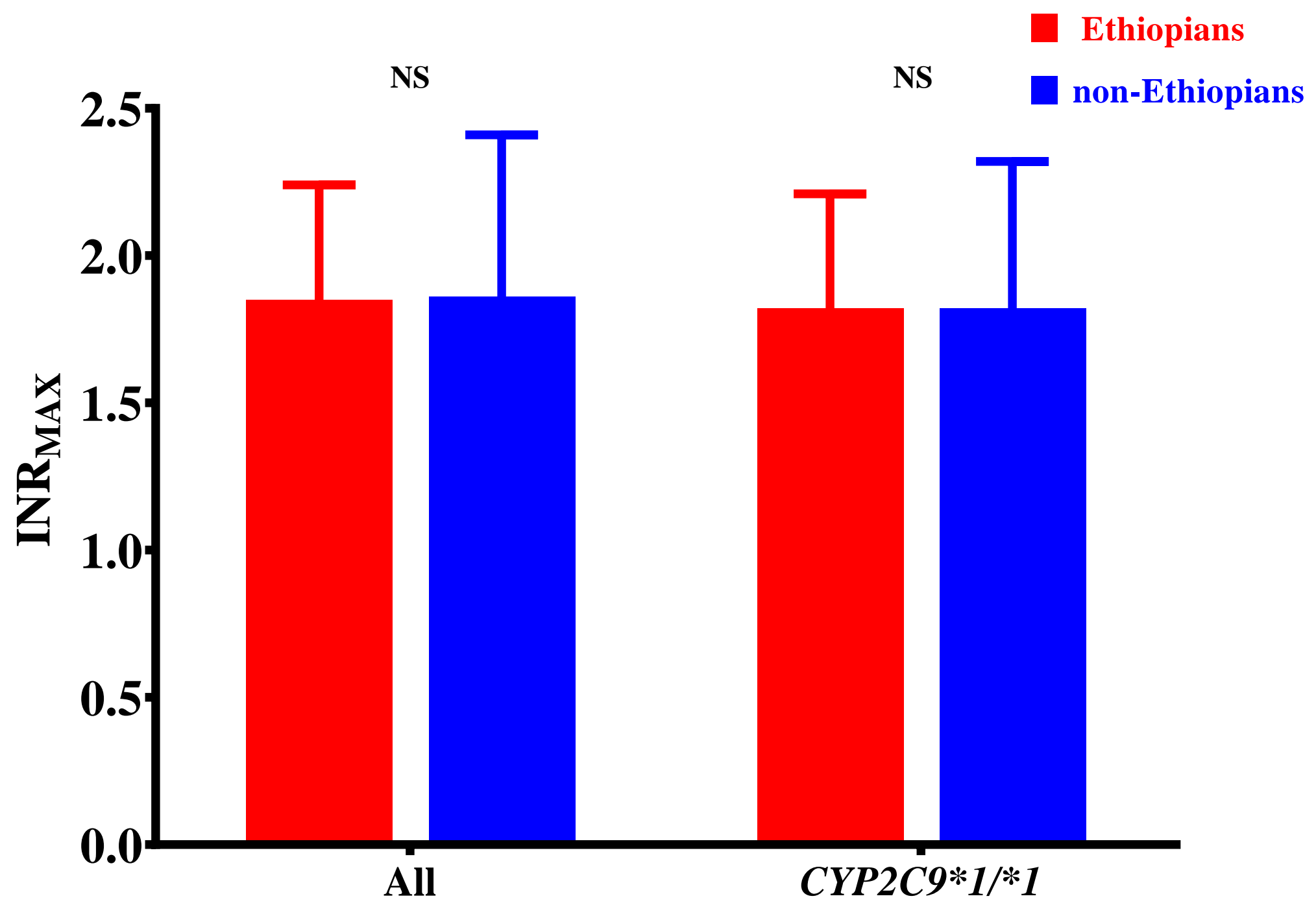

Supplement: Supplementary file 3 [file Image3.pdf]

**Fig. S1: (*R*)-Warfarin Clearance in Ethiopians and non-Ethiopians**

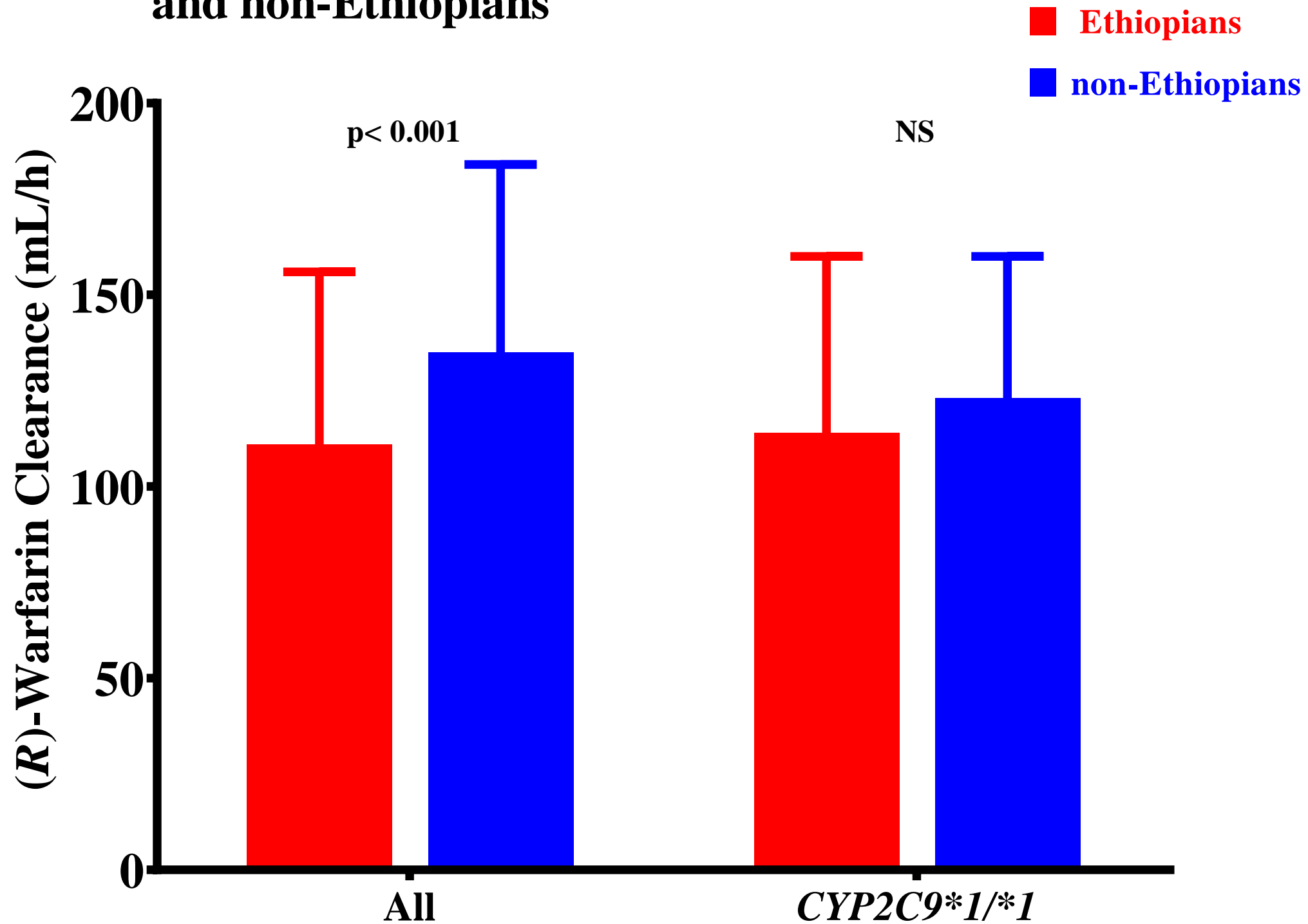

Supplement: Supplementary file 5 [file Image1.pdf]
