# Supplementary material for: Comparison of CYP2C9 activity between Ethiopian and non-Ethiopian Jews: an interethnic study of (S)-warfarin pharmacokinetic and pharmacodynamic
Source: Front Pharmacol. 2026 Jun 23;17:1836874. doi: 10.3389/fphar.2026.1836874 (PMC13337887; doi:10.3389/fphar.2026.1836874)
Supplement: Supplementary file 4 [file Table2.docx]

Table S2: Comparison of AUCINR_120_ and INR_MAX_ between Ethiopians and non-Ethiopians in carriers of *CYP2C9*1/*1* genotype and different *VKORC1* and D36Y genotypes.

|  |  | Ethiopians  (N) | Non-Ethiopians  (N) | p value |
| --- | --- | --- | --- | --- |
| AUCINR_120_ (hours) |  |  |  |  |
|  | *CYP2C9*1/*1* | 168 ± 27  (118) | 165 ± 26  (93) | NS |
|  |  |  |  |  |
|  | *CYP2C9*1/*1-VKORC1* |  |  |  |
|  | AA | 210 ± 34  (14) | 189 ± 28  (28) | < 0.03 |
|  | AB | 170 ± 20  (58) | 159 ± 17  (45) | < 0.002 |
|  | BB | 153 ± 18  (46) | 147 ± 13  (20) | < 0.085 |
|  |  |  |  |  |
|  | *CYP2C9*1/*1-D36Y* |  |  |  |
|  | CC | 172 ± 28  (85) | 166 ± (26  (89) | NS |
|  | CA | 157 ± 22  (32) | 157 ± 18  (4) | NS |
|  | AA | 184  (1) | (0) |  |
|  |  |  |  |  |
| INR_MAX_ |  |  |  |  |
|  | *CYP2C9*1/*1* | 1.82 ± 0.39  (118) | 1.82 ± 0.50  (93) | NS |
|  |  |  |  |  |
|  | *CYP2C9*1/*1-VKORC1* |  |  |  |
|  | AA | 2.45 ± 0.42  (14) | 2.30 ± 0.60  (28) | < 0.085 |
|  | AB | 1.85 ± 0.31  (58) | 1.69 ± 0.26  (45) | < 0.004 |
|  | BB | 1.59 ± 0.22  (46) | 1.47 ± 0.17  (20) | < 0.03 |
|  |  |  |  |  |
|  | *CYP2C9*1/*1-D36Y* |  |  |  |
|  | CC | 1.87 ± 0.40  (85) | 1.83 ± 0.50  (89) | NS |
|  | CA | 1.68 ± 0.34  (32) | 1.64 ± 0.24  (4) | NS |
|  | AA | 1.89  (1) | (0) |  |
